# Thiyl Radical Reactions in the Chemical Degradation of Pharmaceutical Proteins

**DOI:** 10.3390/molecules24234357

**Published:** 2019-11-28

**Authors:** Christian Schöneich

**Affiliations:** Department of Pharmaceutical Chemistry, University of Kansas, 2093 Constant Avenue, Lawrence, KS 66047, USA; schoneic@ku.edu; Tel.: +1-785-864-4880

**Keywords:** protein stability, therapeutic proteins, thiyl radicals, oxidation, fragmentation, cross-link

## Abstract

Free radical pathways play a major role in the degradation of protein pharmaceuticals. Inspired by biochemical reactions carried out by thiyl radicals in various enzymatic processes, this review focuses on the role of thiyl radicals in pharmaceutical protein degradation through hydrogen atom transfer, electron transfer, and addition reactions. These processes can lead to the epimerization of amino acids, as well as the formation of various cleavage products and cross-links. Examples are presented for human insulin, human and mouse growth hormone, and monoclonal antibodies.

## 1. Introduction

The physical and chemical stability of proteins are critical for the efficacy and safety of protein therapeutics [1,2]. The reactions of free radicals play an important role in the chemical degradation of peptide and protein therapeutics in pharmaceutical formulations. For example, pharmaceutical excipients, such as polysorbate, are prone to generate peroxyl radicals [3]. Peroxyl radicals (and secondary oxidants derived from peroxyl radicals, such as alkoxyl radicals or hydroperoxides) can oxidize proteins via various pathways [4,5,6], generating a manifold of radical and non-radical intermediates and products. Free radicals can also be generated in pharmaceutical formulations by mechanical shock [7], leading to cavitation, exposure to light [8,9,10,11], or ionizing radiation (e.g., during sterilization) [12]. This article focuses on the role of a specific type of radical, the thiyl radical (RS^•^), in the degradation of therapeutic proteins, which is primarily induced by exposure to light.

Photochemically, protein thiyl radicals can be generated through the direct homolytic cleavage of a disulfide bond [13] or via the one-electron reduction of a disulfide bond [14,15] (here, we do not consider thiyl radical generation through the oxidation of thiols, as pharmaceutical proteins rarely contain free thiols). The direct photochemical cleavage of disulfides may be relevant for specific conditions, e.g., when chromatographic protein separations are monitored by UV detection (e.g., by UV-C light; λ ≤ 280 nm) or if protein preparations are exposed to UV-C light for viral decontamination [16]. However, photo-induced electron transfer, e.g., from Trp to disulfide, occurs during exposure to UV-B light (λ = 280–315 nm) [9,17], as well as exposure to photostability testing conditions according to the International Conference on Harmonization (ICH), guideline ICHQ1B [10,11]. Importantly, in the presence of high concentrations of salt, even the exposure to visible light results in the photo-ionization of Trp [18].

Thiyl radicals can engage in a great variety of reactions including hydrogen atom transfer (HAT), electron transfer (ET), and addition/elimination reactions. In this way, they can react with literally all of the 20 essential amino acids, though rate constants, e.g., for HAT reactions, may vary with amino acid structure [19]. Important information about the potential reactions of thiyl radicals in proteins can be gleaned from enzymatic processes, rendering at least some thiyl radical-mediated degradation pathways of pharmaceutical proteins “biomimetic” [20].

Thiyl radicals can also indirectly affect protein structure and function, e.g., via the non-enzymatic chemical transformations of mono- and polyunsaturated fatty acids. These transformation reactions can involve oxidation [21,22] and cis/trans-isomerization [22,23,24]. Specifically, thiyl radicals, which can partition into the lipid environment, induce the cis/trans-isomerization of unsaturated fatty acids in biological membranes, e.g., the HS^•^ radical [25] (derived from H_2_S/HS^−^) or the HO-CH_2_CH_2_S^•^ radical [26] (derived from 2-mercaptoethanol). On the other hand, thiyl radicals from glutathione (GSH) show little efficiency in the cis/trans isomerization processes of biological membranes due to their hydrophilicity [26]. Changes in lipid structure through oxidation can promote the conformational changes of polypeptides and proteins, e.g., of amyloid-beta [27]—the main component of amyloid plaques present in Alzheimer’s disease brains [28]. Lipid peroxidation products chemically modify proteins [29]. Moreover, the presence of trans fatty acids in membranes can modulate the intramembrane proteolysis of the amyloid precursor protein (APP) [30], leading to an enhanced generation of amyloid-beta.

In the following, we provide a general overview on the free radical reactions of thiyl radicals that are relevant for the degradation of proteins and, subsequently, summarize recent results on individual pharmaceutical proteins.

## 2. Thiyl Radicals in Reversible HAT Reactions

Thiyl radicals engage in reversible HAT reactions, either inter- or intramolecularly. Early results by Walling and Rabinovitz on product formation during the reaction of isobutylthiyl radicals [2-mythylpropane-1-thiyl radicals; (CH_3_)_2_CH-CH_2_-S^•^)] with cumene suggested the reversibility of Reaction (1) [31].


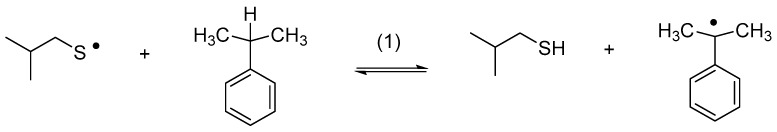


Relative rate constants for the reaction of cyclohexanethiyl and benzenethiyl radicals with a number of substrates, including cumene, were subsequently provided by Pryor et al. [32,33]. In an elegant study, Akhlaq et al. demonstrated that the exposure of 2,5-dimethyltetrahydrofurane to thiyl radicals resulted in cis/trans isomerization (Reactions (2) and (3)) via a chain reaction, a process from which k_2_ and k_−3_ were derived as ca. 10^4^ M^−1^s^−1^ [34]. Similar rate constants were later measured by pulse radiolysis for the reactions of various thiyl radicals with aliphatic alcohols and ethers [35,36] and by a kinetic NMR method for the reaction of thiyl radicals with carbohydrates [37].


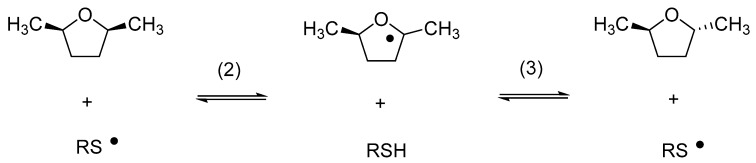


In synthetic procedures, thiols are employed as so-called “polarity-reversal” catalysts [38,39], due to the propensity of thiyl radicals to react via HAT with a series of organic substrates such as alcohols, ethers and amines.


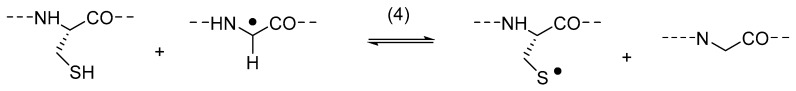


The reversibility of HAT between thiyl radicals and amino acids is of significance for glycyl radical enzymes (GRE), such as ribonucleotide reductase (RNR), pyruvate formate lyase (PFL), glycerol dehydrogenase, benzylsuccinate synthase, and 4-hydroxyphenylacetate decarboxylase [40]. In these enzymes, active site Cys thiyl radicals (CysS^•^) are generated by HAT to glycyl radicals (Gly^•^) (Reaction (4)), and Gly^•^ can be restored by the reverse reaction (Reaction (-4)).


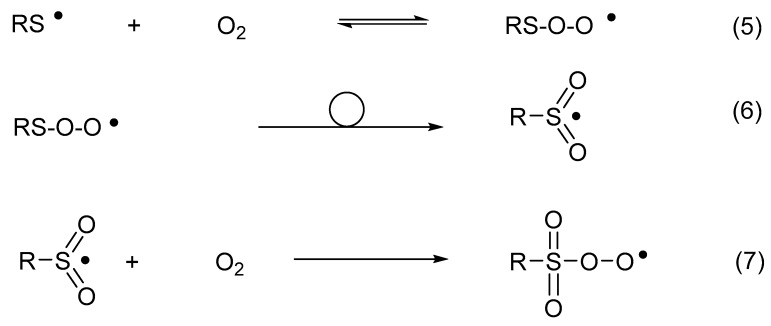


The location of Equilibrium (4) is controlled by conformational properties of Gly^•^ within the protein environment, illustrated here for the case of PFL. Electron paramagnetic resonance (EPR) spectroscopy has demonstrated that the active form of PFL harbors a Gly^•^ radical at the Gly^734^ position, where hyperfine coupling constants indicate that Gly^•^ adopts a planar conformation. This Gly^•^ radical exchanges its α-hydrogen with the solvent via a HAT reaction with Cys [41]. In the planar conformation, however, Gly^•^ is more stable than CysS^•^, based on a ca. 3.4 kcal/mol lower ^α^C–H bond energy of Gly as compared to the S–H bond energy of Cys; as such, Equilibrium (4) is located on the site of Gly^•^ [42]. In order to afford HAT from Cys to Gly^•^, generating CysS^•^, Gly^•^ has been proposed to adopt a less planar conformation, as supported by its location within the protein framework, rendering Gly^•^ 4.6 kcal/mol less stable than CysS^•^ and moving Equilibrium (4) towards the site of CysS^•^ [42]. Theoretical calculations by Rauk and coworkers suggested that HAT reactions occur between thiyl radicals and the ^α^C–H bonds of amino acids located in random and β-sheet conformations, but these do not occur when amino acids are located in α-helices [43]. Experimentally, the inter- and intramolecular HAT reactions of thiyl radicals have been demonstrated for amino acids, amino acid derivatives and peptides, including glutathione, in solution and in the gas phase [19,44,45,46,47,48,49,50]. Importantly, these HAT reactions do not only target ^α^C–H bonds but also C–H bonds of amino acid side chains [51,52]. Together, the experimental data and theoretical calculations on biologically significant HAT reactions in GRE and HAT reactions in amino acids, amino acid derivatives [45,53], and peptides inspired us to consider the possibility of thiyl radical-dependent HAT processes and other reactions in the degradation of protein therapeutics.

## 3. Thiyl Radical Reactions with Molecular Oxygen 

Thiyl radicals reversibly add oxygen to yield thiylperoxyl radicals (RSOO^•^) (Reaction (5)) [54]. The latter can rearrange to sulfonyl radicals (RS^•^O_2_) (Reaction (6)) [54] and further convert into sulfonylperoxyl radicals (RSO_2_OO^•^) (Reaction (7)) [55]. In the presence of electron or hydrogen donors, sulfonyl radicals convert into sulfonates, while sulfonylperoxyl radicals ultimately yield sulfonates [56,57].

## 4. Insulin

### 4.1. HAT Reactions in Solution

**Figure 1:**
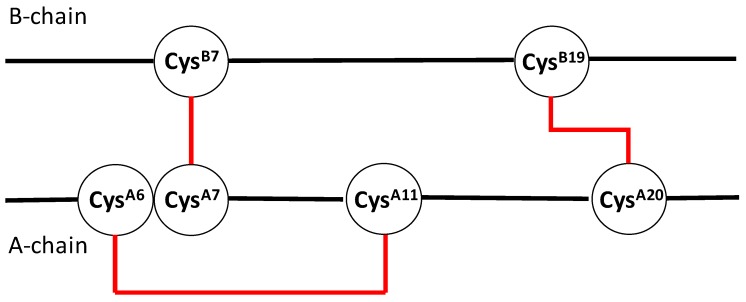
Cartoon displaying the disulfide bonds (in red) of insulin.

Insulin is a small protein containing two separate chains (A- and B-chain), connected by two interchain disulfide bonds (Cys^A7^–Cys^B7^ and Cys^A20^–Cys^B19^) [58]. A third, intrachain disulfide bond connects Cys^A6^ and Cys^A11^ [58]. The disulfide bonds of insulin are shown in the cartoon in Figure 1.

It is well known that insulin is sensitive to chemical and physical degradation, such as the photolytic cleavage of disulfide bonds [59,60] and dityrosine formation [60], deamidation [58,61,62], and fibrillation [58,63,64]. The biologically active form of insulin is the monomer, which exists at insulin concentrations <0.1 μM in the absence of Zn^2+^ [58]. At higher concentrations, insulin exists as a dimer [58], where self-association specifically involves the B8, B9, B12, B13, B16, and B23–28 residues [65].

Important for dimer formation is an aromatic triplet, consisting of the residues Phe^B24^, Phe^B25^, and Tyr^B26^, which is part of an antiparallel β-sheet present at the dimer interface [66]. This dimer interface is different from the sequences responsible for fibrillation [63,64], mainly L^B11^VEALYL^B17^, causing the formation of the cross-β spine motif. The Phe^B24^ residue is equally important for the binding of insulin to the insulin receptor [66]. Interestingly, the substitution of l-Phe^B24^ by d-Phe^B24^ caused a significant increase of insulin affinity to the insulin receptor [67]. Therefore, it was of interest to evaluate whether thiyl radical-mediated intramolecular HAT reactions would proceed in insulin, whether such reactions would be restricted to specific amino acid residues, and whether these would include Phe^B24^, possibly converting l-Phe^B24^ into d-Phe^B24^.

**Scheme 1:**
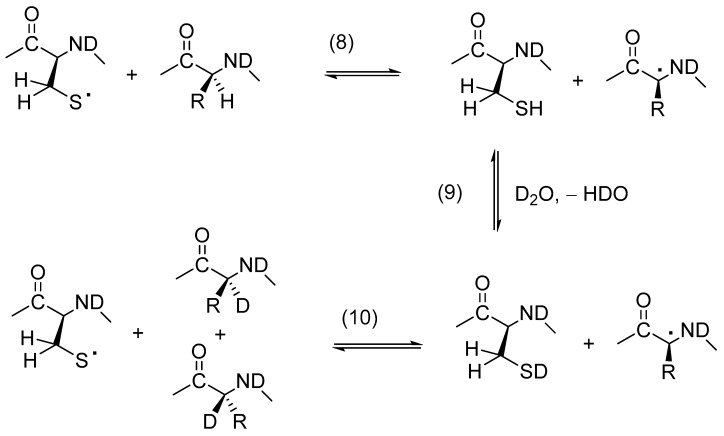
Covalent H/D-exchange mediated by thiyl radicals, representatively shown for an ^α^C–H bond.

The potential for insulin-derived CysS^•^ to engage in intramolecular HAT reactions was monitored by covalent H/D-exchange according to the general Reactions (8)–(10) in Scheme 1, which representatively show the HAT of amino acid ^α^C–H bonds. Solutions of Zn^2+^-free insulin (50 or 500 μM) in either H_2_O or D_2_O were exposed to UV photolysis at 253.7 nm, followed by the alkylation of Cys and the HPLC-MS/MS analysis of an endoproteinase GluC-derived peptide map.

The predominant site of the covalent H/D exchange in the A-chain was Cys^A20^, confirmed by the MS/MS sequencing of the Asn^A18^–Tyr–Cys–Asn^A21^ peptide after photolysis in D_2_O. This Cys residue is located at the end of the α-helix formed between the A13 and A20 residues. On the B-chain, the covalent H/D-exchange was most prevalent between the Leu^B6^ and Ser^B9^ residues and between the Val^B18^ and Gly^B20^ residues. Hence, deuterium incorporation proceeded selectively and did not target Phe^B24^, suggesting that Phe^B24^ is also not a target for thiyl radical-mediated epimerization. Based on the calculations by Rauk et al. [43], the lack of a covalent H/D exchange at the ^α^C–H bond of Phe^B24^ cannot be rationalized with an effect of the antiparallel β-sheet structure around the aromatic triplet (Phe^B24^–Phe^B25^–Tyr^B26^) in the insulin dimer on the ^α^C–H bond energy of Phe^B24^. However, it is possible that the protein conformation did not permit the reaction of any of the photolytically generated CysS^•^ radicals with Phe^B24^, which would have excluded a covalent H/D exchange at both the ^α^C–H and ^β^C–H bonds of Phe^B24^. Importantly, a covalent H/D-exchange occurred either in the vicinity of Cys or on Cys itself. This result is consistent with studies on small Cys-containing model peptides where deuterium incorporation has been found to be most efficient at residues −1 or +1 from Cys. Deuterium incorporation into Cys itself is consistent with the 1,2- and 1,3-HAT reactions of thiyl radicals [45,53], for which rate constants were recently reported [45]. Additional evidence for the 1,2-HAT reactions of CysS^•^ radicals in proteins comes from studies with *Escherichia coli* class III ribonucleotide reductase, where electron spin resonance (ESR) studies revealed the presence of an H/D exchange at the ^β^C–H bond of a CysS^•^ radical [68].

An interesting product detected by MS/MS analysis was a cross-link between Tyr^A19^ and Cys^B20^. This cross-link can form by the reaction of a CysS^•^ radical with a tyrosyl radical (TyrO^•^). Under our experimental conditions, a pair of TyrO^•^ and CysS^•^ radicals could be formed via at least two different ways: (i) photo-induced electron transfer from Tyr to cystine, followed by combination of TyrO^•^ and CysS^•^; and/or (ii) the homolytic cleavage of cystine, followed by electron/hydrogen transfer from Tyr to one CysS^•^ radical and a combination of TyrO^•^ with the second CysS^•^ radical. An alternative pathway for the formation of Cys–Tyr cross-links would be the addition of a CysS^•^ radical to Tyr, followed by the oxidation of this radical adduct. In fact, the potential for an addition of CysS^•^ to aromatic amino acids was experimentally and theoretically demonstrated for the reaction of CysS^•^ with Phe. More recently, the fast reversible additions of various radicals to the aromatic amino acid His have been reported [69,70].

### 4.2. Additional Reactions of Thiyl Radicals Leading to Cross-Links in Solution

**Scheme 2:**
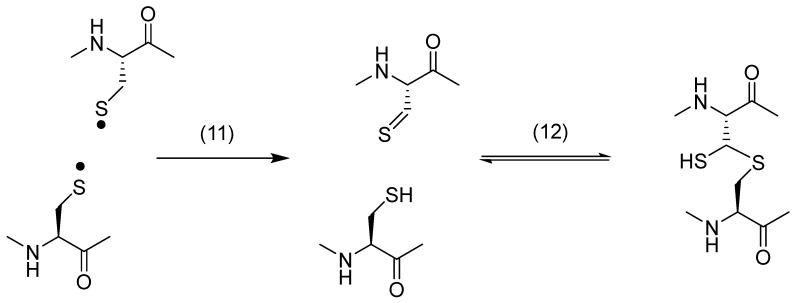
Formation of dithiohemiacetal subsequent to the disproportionation of a thiyl radical pair.

Along with the Cys–Tyr cross-link, the photo-irradiation of insulin in solution generated a dithiohemiacetal cross-link between Cys^A20^ and Cys^B19^ [71]. Such photolytically generated dithiohemiacetal cross-links have also been identified and characterized for various disulfide-containing model peptides and proteins, including human and mouse growth hormone and monoclonal antibodies (see below). Mechanistically, the formation of dithiohemiacetal likely involves the light-induced homolysis of cystine, yielding a CysS^•^ radical pair, which disproportionates to thiol and thioaldehyde, followed by the addition of the thiol to the thioaldehyde (Scheme 2).

### 4.3. Thiyl Radical Reactions in Solids

In order to evaluate the propensity for HAT reactions in solid insulin formulations, we prepared amorphous, crystalline, and microcrystalline human insulin [71]. Photo-irradiation at λ = 253.7 nm yielded a dithiohemiacetal between Cys^A20^ and Cys^B19^, as well as peptide products with reduced Cys at the Cys^B7^ and Cys^B19^ positions, as characterized by HPLC-MS/MS. The photolysis of an amorphous insulin sample, generated by drying a D_2_O solution of insulin, showed no evidence of a covalent H/D exchange, suggesting that the reversible HAT reactions shown in Scheme 1 may not occur to a significant extent in insulin solids. We note, however, that the lack of a covalent H/D exchange at C–H bonds may either be caused by the absence of HAT reactions or by an inefficient H/D exchange of the sulfhydryl group (Scheme 1; Reaction (9)) in solid formulations.

## 5. Growth Hormone

**Figure 2:**
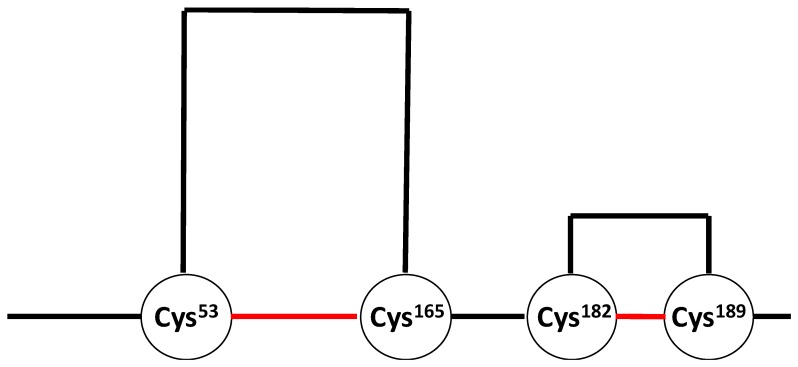
Cartoon displaying the disulfide bonds (in red) of human growth hormone.

Human growth hormone (hGH) belongs to the class of four-helix bundle proteins [72] and is used for the treatment of pediatric hypopituitary dwarfism [73], as well as children [73] and adults [74] with hGH deficiencies. HGH is sensitive to deamidation [73,75,76], N-terminal truncation [77], oxidation [4,7,73,75,76,78,79,80,81], aggregation [73], and photo-degradation [82,83,84]. The structures of a trisulfide [76,85,86,87] and a thioether [88] variant, originating from the biosynthetic pathway, have been characterized by mass spectrometry. HGH contains two disulfides between Cys^53^ and Cys^165^ and between Cys^182^ and Cys^189^ [73]. The Cys^182^–Cys^189^ disulfide bond defines the small C-terminal loop. A cartoon displaying the disulfide bonds of hGH is shown in Figure 2. Mutants of hGH, in which either Cys^182^ or Cys^189^ or both Cys residues are replaced with Ala, show a significantly reduced binding to the human growth hormone receptor [89].

The photolysis of hGH with UV light has resulted in a large number of products originating from the disulfide cleavage and subsequent reactions of CysS^•^ radicals [84]. For example, reduced Cys and thioaldehyde were detected for all Cys residues originally present in the disulfide bonds, and a dithiohemiacetal was formed between Cys^182^ and Cys^189^. In addition, sulfinic and/or sulfonic acid were detected as products of Cys^165^, Cys^182^, and Cys^189^. These oxyacid products are expected from thiyl radicals generated in the presence of oxygen, as described in Section 3. The following discussion shall focus on a few rather unusual degradation products and cross-links, as well as the proposed mechanisms for their formation. In this discussion, the chemical names for structures are given as if they were present in amino acid form (rather than in the protein).

### 5.1. The Conversion of Cys to Gly

**Scheme 3:**
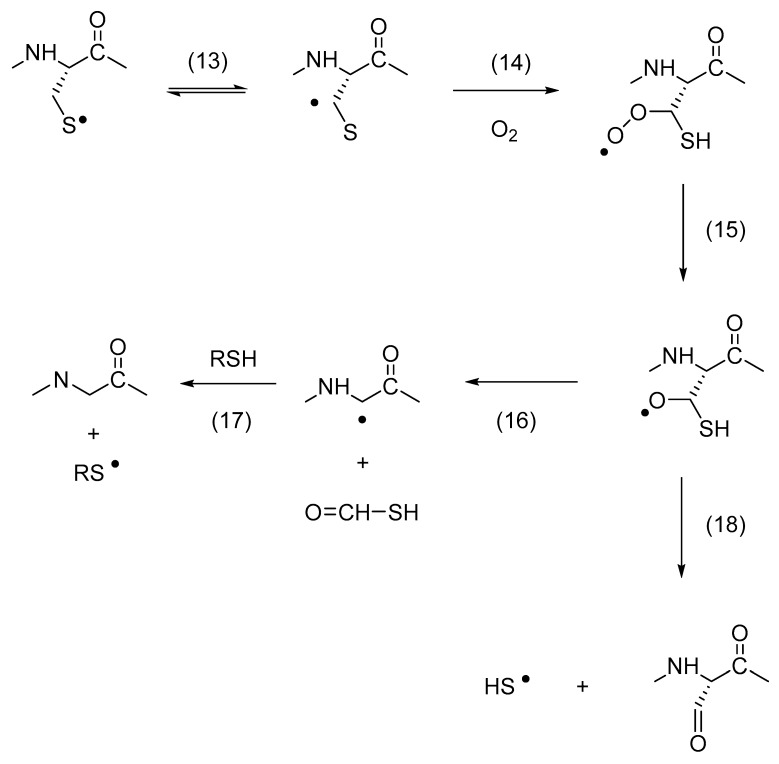
Thiyl radical mediated conversion of Cys to Gly (glycyl).

The conversion of Cys to Gly was detected for Cys^165^, Cys^182^, and Cys^189^; at the same time, Cys^165^ and Cys^189^ were also converted to serine semialdehyde (2-amino-3-oxopropanoic acid; 3-oxoalanine; 2-formylglycine) and Ser. The proposed mechanism for the conversion of Cys to Gly (and serine semialdehyde) is shown in Scheme 3, where a 1,2-HAT reaction [45] of a CysS^•^ radical is critical for the formation of a carbon-centered radical at C_β_, followed by the addition of oxygen to yield a peroxyl radical. A series of reactions could transform the peroxyl radical into an alkoxyl radical, such as oxygen transfer reactions or reactions with additional peroxyl radicals. The alkoxyl radical is precursor for a carbon–carbon bond cleavage—yielding a Gly^•^ radical—or a carbon–sulfur bond cleavage—yielding serine semialdehyde (though we note that serine semialdehyde can also be formed by hydrolysis of a Cys thioaldehyde). The proposal of a 1,2-HAT reaction for a protein CysS^•^ radical also suggests that products of a 1,3-HAT reaction might be observed. In fact, for all hGH Cys residues, the formation of dehydroalanine (Dha) was detected. Dha can form via a 1,3-HAT reaction, followed by the elimination of HS^•^ [45] (though care has to be taken during sample preparation for HPLC-MS/MS analysis, as Dha can also form during proteolytic digestion [90]).

### 5.2. The Formation of Ether and Vinyl ether

The exposure of mouse growth hormone (mGH) to UV light triggered the formation of particles of various sizes [91]. The injection of UV-exposed mGH into Balb/c or nude Balb/c mice caused an immune response, generating antibodies that cross-reacted with unmodified mGH [91]. The mass spectrometry analysis of UV-exposed mGH tentatively revealed the presence of chemical cross-links containing an ether bond between the original Cys^78^ and Ser^188^ residues or a thioether bond between the original Cys^78^ and Cys^189^ residues [91]. Whether these cross-links contribute to the immunogenicity of mGH remains to be shown. However, especially the photochemical conversion of a disulfide into an ether bond involving a neighboring Ser residue, is mechanistically intriguing.

**Scheme 4:**
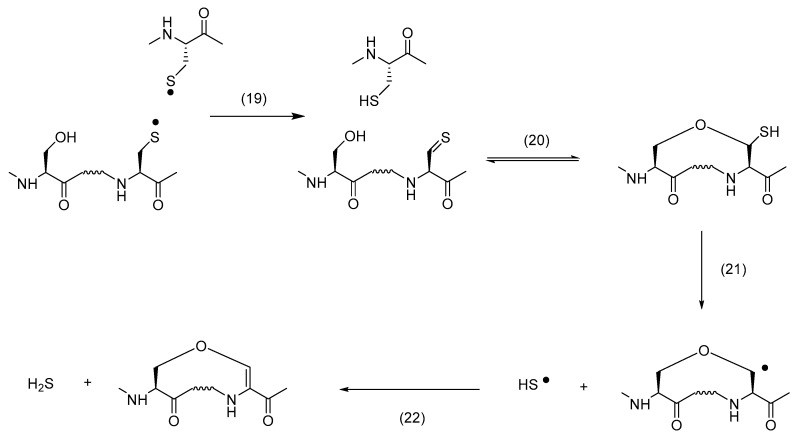
Formation of vinylether from Ser.

**Scheme 5:**
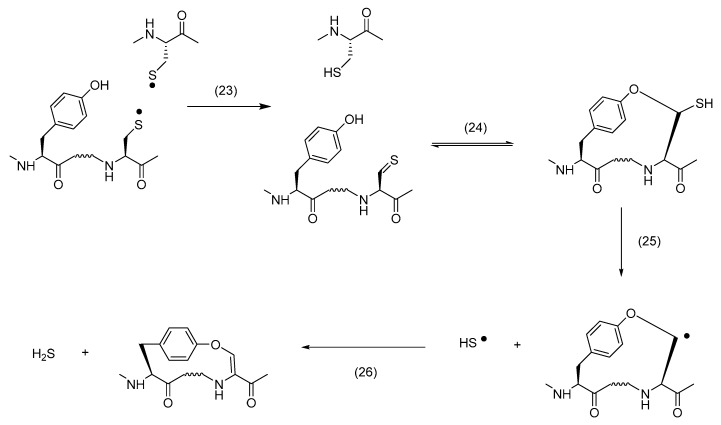
Formation of vinylether form Tyr.

When hGH was exposed to UV light, mass spectrometry analysis revealed the formation of vinyl ether between the original Cys^189^ and Ser^184^ residues and between the original Cys^160^ or Cys^165^ and Tyr^164^ residues [84]. The proposed mechanisms for the formation of these products are displayed in Scheme 4 and Scheme 5, respectively. Key to product formation is the homolytic cleavage of a disulfide bond into a pair of CysS^•^, followed by disproportionation into thiol and thioaldehyde. The latter can react with the hydroxyl group of either Ser or Tyr to yield a thiohemiacetal. Under continuous UV exposure, the C–S bond of the thiohemiacetal is expected to cleave, either homolytically or heterolytically [92,93,94], and the vinyl ether is generated via subsequent oxidation and deprotonation, respectively [95]. An important difference between the results of both growth hormones is the formation of an ether cross-link in mGH vs. a vinylether cross-link in hGH. This may be rationalized by sequence differences between hGH and mGH [96], as we also observed significant differences in photooxidation between hGH and rat growth hormone (rGH) [97]. We believe that the ether cross-link is ultimately generated via reduction of a vinyl ether. The UV exposure of disulfide-containing peptides leads to the formation of Cys [95,98] and H_2_S [95]. Under UV-exposure, the specifically thiolate forms of Cys and H_2_S, i.e., CysS^−^ and HS^−^, release an electron, which can reduce Dha. The latter was experimentally tested during the photo-irradiation of a disulfide-containing model peptide in the absence and presence of methylene chloride (CH_2_Cl_2_), a prominent scavenger of hydrated electrons [99]. Hence, we propose that vinyl ether reduction by a hydrated electron, followed by a HAT—likely from a photochemically generated thiol [95,98]—is key to the generation of an ether cross-link. We observed an analogous mechanism for the formation of a thioether cross-link from vinyl thioether, where CH_2_Cl_2_ inhibited thioether formation during UV-exposure [95]. Interestingly, the UV exposure of hGH also led to both vinyl thioether and thioether cross-links between the original Cys^182^ and Cys^189^ residues [84], i.e., Cys residues which originally form the disulfide bond characterizing the small C-terminal loop of hGH.

### 5.3. Formation of Non-Native Disulfides

The UV exposure of hGH produced significant yields of non-native disulfide bonds, i.e., intramolecularly between Cys^189^ and Cys^189^, as well as either inter-or intramolecularly between Cys^53^ and Cys^182^, and Cys^165^ and Cys^189^ [84]. These disulfide bonds can form via homolytic substitution, i.e., an S_H_2 mechanism that involves the reaction of a thiyl radical with a disulfide bond [100], or via the recombination of two thiyl radicals. In addition, non-native disulfides may form via the reaction of free thiols with disulfide bonds. Free thiols are generated together with thiyl radicals by the one-electron reduction of disulfides. Usually, free thiols are derivatized by alkylation prior to mass spectrometric analysis. However, free thiols can react with disulfide bonds during the time of UV exposure or any other time required for sample preparation; for example, in the case of hGH, sample preparation involved 30 min of reduction of the remaining disulfide bonds at pH 7.5 and 45 °C. The fact that non-native disulfide bonds between Cys^189^ and Cys^189^ and between Cys^53^ and Cys^182^ were not formed in non-photolyzed control solutions of hGH [84] is consistent with a free radical mechanism of disulfide scrambling.

### 5.4. hGH Cleavage Products

The UV-exposure of hGH results in several backbone cleavage products originating from ^α^C^•^ radicals generated at Cys residues or at amino acid residues in the vicinity of Cys residues, e.g., at Cys^53^, Cys^165^ and Leu^52^ [84]. Mechanistically, these fragmentation products are generated through the well-established diamide or α-amidation pathways. What is of interest here is that these fragmentation products again give testimony to the ability of CysS^•^ radicals to generate ^α^C^•^ radicals via intramolecular HAT reactions.

## 6. Monoclonal Antibodies

While a comprehensive product analysis, such as performed for hGH [84], has not yet been completed for monoclonal antibodies, certain products that are analogous to those generated from insulin or hGH have been detected. For example, the UV exposure of an immunoglobulin 1 (IgG1) resulted in the formation of dithiohemiacetal and thioether cross-links [101]. The light exposure of an IgG1 in an Atlas Suntest CPS+ Xenon test instrument, utilized for photostability studies according to the ICHQ1B guideline, resulted in disulfide scrambling [11]. The reactions leading to the formation of non-native disulfides in the IgG1 molecule are likely analogous to those described for hGH in Section 5.3.

The potential of CysS^•^ radicals to engage in intramolecular HAT reactions in monoclonal antibodies was first demonstrated by a UV light-induced covalent H/D exchange analogous to the reactions presented in Scheme 1 [102]. Subsequent studies on an IgG1 revealed that these HAT reactions have the potential to epimerize amino acids in a protein, e.g., to convert L- into D-amino acids [103] (analogous experiments with model peptides [104] and octreotide [105] had demonstrated the ability of CysS^•^ radicals to epimerize amino acids in peptides). Overall, the exposure of an IgG1 to UV light resulted in the generation of D-Glu and D-Val (and some D-Ala), where the relative yields of D-amino acids depended on the presence of various excipients in these formulations [103]. Experimentally, D-amino acids were recovered from the protein by controlled acid hydrolysis, which converts D-Gln to D-Glu, so that the yields of D-Glu were representative for the combined yields of D-Glu and D-Gln generated by HAT. Through proteolytic digestion, peptide fractionation, and the controlled acid hydrolysis of individual peptides, some locations of D-amino acids were identified such as the heavy chain (HC) sequences HC51–59 and HC287–296. Specifically, the sequence HC51–59 is located in the hypervariable region that is responsible for antigen binding, where conformational changes induced by amino acid epimerization may have biological consequences. We note that the exposure of monoclonal antibodies to light results in aggregation [106,107] and immunogenicity [108]. An important study was able to correlate immunogenicity with the presence of chemical modifications on subvisible particles, while particles not carrying chemical modifications were not found to be immunogenic [109].

## 7. Conclusions

The preceding sections provide examples for a variety of mechanisms by which thiyl radicals engage in the chemical degradation of pharmaceutical proteins. Noteworthy are the cross-links generated between thiol oxidation products and either Ser or Tyr. The exposure of the small (ca. 22 kDa) protein human growth hormone yielded nearly 60 different products that originated from thiyl radical generation, and a significantly higher number of products may be expected from the light-exposure of a monoclonal antibody. Comprehensive product studies on monoclonal antibodies are ongoing in our laboratory and will be reported in due time.
